# CRISPR-Associated (CAS) Effectors Delivery via Microfluidic Cell-Deformation Chip

**DOI:** 10.3390/ma14123164

**Published:** 2021-06-09

**Authors:** Noshad Peyravian, Maziar Malekzadeh Kebria, Jafar Kiani, Peiman Brouki Milan, Masoud Mozafari

**Affiliations:** 1 Cellular and Molecular Research Center, Iran University of Medical Sciences, Tehran 1449614535, Iran; n.peyravian@gmail.com (N.P.); mazi.malekzadeh@gmail.com (M.M.K.); 2 Department of Tissue Engineering and Regenerative Medicine, Faculty of Advanced Technologies in Medicine, Iran University of Medical Sciences, Tehran 1449614535, Iran; 3 Department of Molecular Medicine, Faculty of Advanced Technologies in Medicine, Iran University of Medical Sciences, Tehran 1449614535, Iran; ja.kiani@gmail.com; 4 Oncopathology Research Center, Iran University of Medical Sciences, Tehran 1449614535, Iran

**Keywords:** CRISPR, microfluidics, genome, Cas9 protein, tissue engineering

## Abstract

Identifying new and even more precise technologies for modifying and manipulating selectively specific genes has provided a powerful tool for characterizing gene functions in basic research and potential therapeutics for genome regulation. The rapid development of nuclease-based techniques such as CRISPR/Cas systems has revolutionized new genome engineering and medicine possibilities. Additionally, the appropriate delivery procedures regarding CRISPR/Cas systems are critical, and a large number of previous reviews have focused on the CRISPR/Cas9–12 and 13 delivery methods. Still, despite all efforts, the in vivo delivery of the CAS gene systems remains challenging. The transfection of CRISPR components can often be inefficient when applying conventional delivery tools including viral elements and chemical vectors because of the restricted packaging size and incompetency of some cell types. Therefore, physical methods such as microfluidic systems are more applicable for in vitro delivery. This review focuses on the recent advancements of microfluidic systems to deliver CRISPR/Cas systems in clinical and therapy investigations.

## 1. Introduction

Genome modification including deletion and modification of DNA strands at target points has created high-light apertures to improve clinical therapy. The first clinical trial of gene therapy in children with severe ADA-associated combination immunodeficiency (ADA ID SCID) was injected with T cells, which have shown a modification in the ADA gene [1,2]. Although the first step in integrating transgenes through homologous recombination has been taken, clinical trials in the next 40 years have been altered to apply a new endonuclease-based gene-editing method with higher efficiency. These approaches contain zinc finger nucleases (ZFNs), Effective Nucleases such as Transcription Activators (TALENs), and regular and intermediate palindromic replications CRISPR/Cas endonucleases, which is a cleft DNA endonuclease that recognizes and connects to targeted sequences [3,4] The potential off-target properties of CRISPR/Cas edited human cell lines have been highlighted in several in vitro and in vivo studies [5,6]. The first CRISPR human clinical treatment on CRISPR/Cas9 modified immune cells started at West China Hospital to remedy patients with lung cancer in 2016 [7,8]. Later, a scientist began the birth of CRISPR-edited neonates in humans, simultaneously conducting clinical trials of CRISPR genome-editing therapies in China and the United States for cancer therapy and β-thalassemia, respectively [9].

Although CRISPR technology has indicated elementary success with medical and ethical rigor, delivery within the genome editing operating system remains challenging. Despite viral vectors showing high efficiencies in the delivery and expression of genes, virus transmission techniques are generally concerned about their potential for carcinogenicity and immunogenicity. Recently, advances in non-viral transmission systems have been created with the development of a wide range of carriers such as nano, micro and channels with a diversity in targeting specific changes, so that in cultured cells or in vivo, physical delivery has been used to provide DNA encoding via plasmids [10].

In this review, we focused on examining how to design the CRISPR/Cas gene editing and detection tools and the subsequent delivery of these systems by relying on the microfluidic method to the intended gene population in vitro and/or in vivo studies to attain efficient gene editing for clinical applications.

## 2. Limitations of Previous Automated Delivery Technologies

Although microfluidics can provide the researcher with many advantages, it is not a cure-all. Indeed, many features of microfluidic systems that were once supposed critical to their use or adoption are now less compelling to the attendant improvements and refinements in conventional technologies [11]. In simple terms, a microfluidic tool must make a persuasive case for adoption based on factors such as analytical performance, usability, and information yield. The advantages of transferring macroscale electrophoresis platforms to chip-based formats are undeniable from the point of view, speed, throughput, integration with downstream processing, and analytical efficiency. Many molecular biologists still prefer to pour macroscale gels because these yields essentially give the same information, albeit on longer timescales [12,13].

A detailed assessment of the material limitations on microfluidic technology is well beyond the scope of the current discussion. Despite many efforts, the fabrication of active mechanical components in microfluidic devices consisting of rigid materials remains a difficult, complex, and expensive procedure, hindering the pace of device development. The advent of soft lithography to fabricate devices in the elastomer polydimethylsiloxane (PDMS) catalyzed microfluidic research growth in the early–late 1990s. Indeed, many new laboratories use nothing else because of the low technology and investment threshold required to structure with micrometer-scale resolution [14,15]. Despite its widespread use, PDMS has many limitations and is ill suited to mass production. Conversely, glass or silicon substrates are robust, but require sophisticated fabrication processes whose cost or access is prohibitive to many.

Regarding the fluid manipulation in microfluidics, most suffer from disadvantages such as a dependence on the details of fluid and surface properties, a lack of reconfigurability, or a lack of individual valve control [16].

## 3. Microfluidic Devices for Gene Delivery Systems

Gene delivery is a potential method for treating different diseases including gene-related disorders, infectious diseases, AIDS, and cancer. An important application area for microfluidics is genomics, and in this case is instructive in understanding the drivers of success. Microfluidics provides a natural solution to this problem by miniaturizing and automating nucleic acid biochemistry in high-throughput formats. Small-volume analysis can also fundamentally improve the sensitivity of genomic analysis, enabling experiments with very limited templates. Accordingly, by scaling reactions down to nanoliter or picoliter volumes, microfluidics simultaneously provides throughput and economy while also enhancing performance. Gene delivery is based on the process of introducing into a nucleus an engineered pDNA (plasmid DNA) that encodes a functional, therapeutic gene that helps modulate cellular functions and responses. However, efficient delivery requires the pDNA to be protected; pDNA/cationic liposome (CL) complexation is a promising strategy for non-viral gene therapy where that liposome is produced using microfluidic hydrodynamic focusing devices [17].

Moreover, using microfluidics approaches, three gene transfection techniques have been explored including electric pulse, hydrodynamic force, and optical energy. The microfluidic device by hydrodynamic force has parallel microfluidic channels with micro-holes for trapping single cells [18]. A vector DNA encoding extracellular signal-regulated kinase (ERK1) protein, known for transducing signals from the cell’s environment to the cell nucleus, was transfused into human mesenchymal stem cells (hMSC) [19]. Using the hydrodynamic focusing of the microfluidic device, the delivery of DNA and siRNA into several hard-to-transfect cell lines including Neuro-2A, PC12, and C2C12 cells significantly enhanced the transfection efficiency and viability. In the literature, the inertial vortices in an electroporator effectively mix cells and exogenous DNA. As a result, cells in such a flow field are exposed to a complex combination of transverse advection and rotation. Indeed, a larger cell surface area contacts the exogenous DNA and electric field, resulting in more uniform permeabilization [20,21].

## 4. CRISPR/Cas9 Systems and Designing the sgRNA

The term CRISPR, or dense regular palindromic replicates, describes a new family of DNA replication sequences presented in the prokaryotic genome. Different CRISPR/Cas mediated genome is composed of two classes. Their multiple effector molecules characterize class 1 CRISPR systems. The effector molecules contain complexes that are responsible for RNA recognition and crRNA binding. Class 1 effector molecules are similar between the types, despite their distinct sequences. Class 2 CRISPR systems are characterized by the presence of a single effector molecule. Types I, III, and IV Cas proteins are class 1; however, class 2 includes types II, V, and VI Cas proteins that use distinct genome recognition and depreciation mechanisms. There are seven different subtypes of class 1, type I CRISPR systems. All class 1 type I subtypes contain cas3 loci that can unwind double-strand DNA and RNA–DNA complexes to facilitate target cutting [22,23]. Like type I, type III systems all share a standard cas locus, in this case, cas10. Cas10 encodes something similar to an RNA recognition motif called Palm and a cyclase domain responsible for cutting. Type III systems can recognize both DNA and RNA for cleavage. Type IV is a putative class 1 system, whereby relatively little is known about it compared to types I and III. The most common class 2 system type is type II. Type II systems are characterized by the presence of Cas9 and ancillary proteins cas1 and cas2. Similar to type II systems, type V also requires tracrRNA for function. Type VI is the only class 2 system that targets RNA for editing [23,24]. The modular nature of Cas9 has made it a versatile tool by targeting a nuclease protein to amplify both target DNA strands by guiding RNA sequences. It has been performed in specific nucleic acid locations for focused analysis in human and mouse cell lines [25].

Class 2 type II classification includes the CRISPR/Cas9 system and the system obtained from the Streptococcus pyogenes bacteria, so the most extensively used of the Cas9 proteins is related to this bacteria. Cas9 is a wild-type protein with two nuclear modes, HNH and RuvC, and the targeted activity of Cas9 can be driven by two RNA fragments that form a dual state such as crRNA and CRRNA activator trans (tracrRNA). Activation of the CRISPR system begins with the transcription of repetitive sequences to the CRISPR RNA precursor (pre-crRNA) and is then cleaved into CRRNA by a tracrRNA at its 5 ends with fused CR-ARNA [26]. The adult crRNA-tracrRNA product, using RNase III and cas9, forms an RNA-endonuclease complex consisting of tracrRNA, crRNA, and Cas9. This set demands the DNA sequence to play the abutting Prospasaker (PAM or short DNA sequence) that complements the CRRNA and is recognized by the CRISPR/Cas system. crRNA guides deliver Cas9 to the target sequences, and the combination of crRNA in the complementary defined site as a structural alteration cleaves the target site with activating nuclease domains in Cas9. Cas9 disports the complementary cr-RNA sequence with the HNH domain as well as cleaves the opposite strand and produces its end by a RuvC-like domain.

Wild-type Cas9 and engineered mutations are combined with other functional protein domains as a ligand and provide the ability to design new CRISPR systems that can be turned off, activated, epigenetically modified, enhanced, or even (DNA and RNA) single base editing. Bifurcation can be generated by CRISPR/Cas9-mediated gene modification in two ways: terminal non-homologous binding (NHEJ) and homologous repair (HDR). NHEJ is the dominant repair pathway that tends to make errors, although it is an efficient repair pathway that generates indels, leading to frame change mutations and subsequently dysfunctional genes [27]. In tumorigenesis, the CRISPR/Cas9 system can generate double-strand bounds within the exon sequences of the mutated alleles that NHEJ renovates to create indels that interrupt the oncogenes. The HDR pathway often happens in the form of single-stranded oligodeoxynucleotides (ssODNs) to correct point mutation or the exact integration of a functional site into the genome of the target cells that can be used in gene therapy [28].

The usage of small molecules as an inhibitor factor such as Scr7, shRNA, and proteins that affect DNA ligase IV (essential enzymes in the NHEJ and RS-1 pathways) stimulate the human HR RAD51 protein, rather than mechanisms of DNA repair leading to HDR pathways. Additionally, Cas9 sgRNA and nuclease transition to the late G2 phase of the cell cycle or a combination of Cas9 nuclease to the Geminin protein to utilize the optimal time of homologous repair pathways in the late S and G2 phase [4,29].

One of the requirements for successful modification of the eukaryotic genome using the CRISPR/Cas9 system is the presence of the guide RNA (sgRNA or crRNA/duplex crRNA/tracrRNA) Cas9 protein complex and the introduction of mRNA or DNA. It is a therapeutic approach in which the genome of specific cells is re-transplanted into the patient in vitro, and then the modified cells for therapeutic effect. This method avoids this problem by accurately editing the cello type, creating an opportunity for successful editing screening [30].

## 5. The other CRISPR/Cas Approaches for Diagnosis

In past studies, CRISPR-associated methods have been used to find nucleic acids by using different CRISPR-associated CAS effectors like Cas12 to detect single-stranded DNA (ssDNA). Cas12 is a compact and efficient enzyme that creates staggered cuts in dsDNA. Cas12 processes its guide RNAs, leading to increased multiplexing ability. Cas12 has also been engineered as a platform for epigenome editing, and it was recently discovered that Cas12a can indiscriminately chop up single-stranded DNA once activated by a target DNA molecule matching its spacer sequence. This property makes Cas12a a powerful tool for detecting tiny amounts of target DNA in a mixture [31]. Cas12a nuclease, class II CRISPR/Cas12a type V, is capable of the procedure from pre-crRNA to mature crRNA without the existence of tracrRNA compared to CRISPR/Cas9 systems, thus diminishing the plasmid size. Cas13 is an outlier in the CRISPR world because it targets RNA, and not DNA. Once it is activated by a ssRNA sequence bearing complementarity to its crRNA spacer, it unleashes a nonspecific RNase activity and destroys all nearby RNA regardless of their sequence. The microbial CRISPR effector Cas13a (previously named C2c2) showed the other (Cas) effectors and a triggered cleavage capability of nontarget single-stranded RNAs (ssRNAs) in the surrounding. It was recently used as a genome editing tool, which is considered as a powerful molecular scissor to apply in the genome editing program. This method suggested various other CRISPR/Cas9 or Cas12a applications for genome therapy editing including clinical strategies for human immunodeficiency virus infection and cancer immunotherapy (B-cell leukemia and acute T-cell lymphoblastic leukemia) [32].

**Figure 1:**
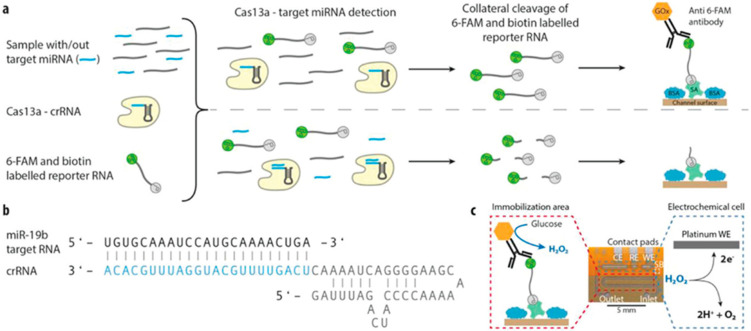
Combination of the CRISPR technology along with an electrochemical microfluidic biosensor for miRNA diagnostics. (**a**) Schematic of the off-chip miRNA targeting including the enzyme Cas13a, the target miRNA (blue), the target-specific crRNA, and the biotin and 6-FAM-labeled reporter RNA, which is immobilized after the cleavage process onto the streptavidin (SA) and BSA blocked channel surface. (**b**) Schematic of the single-stranded target miRNA, miR-19b, and the crRNA, where the complementary sequence is highlighted in blue. (**c**) Working principle and photo of the microfluidic biosensor with its main elements including the contact pads for the working, reference, and counter electrode (WE, RE, CE) in the electrochemical cell (marked in blue) and the immobilization area for the assay preparation (highlighted in red), separated by the hydrophobic stopping barrier SB, reproduced with permission from [36] Copyright 2020 Wiley.

Cas13a, Cas13b, Cas13c, and Cas13d are four subtypes of the massive Cas13 family that can bind and degrade RNA in a programmed manner, shield bacteria from RNA phages, and function as a substrate. Additionally, regular cross-linked palindromic repetitions (CRISPR)-Cas13a, formerly known as CRISPR-C2c2, is the recently identified CRISPR-Cas RNA system RNA with unique single-strand characteristics to manipulate RNA [33]. RNA degradation activity (ssRNA) destroys nearby RNAs, regardless of their sequence. When coroa binds to a CRISPR-RNA (crRNA), it forms an effective set of effectors targeting CRRNA-guided RNA. Cas13a is the newest CRISPR-Cas system, belonging to the Class 2 system and type VI. In addition, for the first time in Leptotrichia Shahii, anaerobic Gram-negative rod bacteria were identified as part of the natural oral and intestinal flora [34,35]. Gootenberg J et al. incorporated the parallel effect of an ortholog of Cas13a from Leptotrichia Wadei with recombinase polymerase amplification (RPA) that can be coupled with T7 transcription to convert amplified DNA to RNA for detection by LwCas13a as well as isothermal amplification to establish a CRISPR-based diagnostic (CRISPR-Dx), providing rapid DNA or RNA detection with attomolar sensitivity and single-base mismatch specificity [36,37] (see Figure 1).

Although the CRISPR/Cas9 system has been developed for several disease models, there are several challenges to translating this technology. The main factors are needed to be identified to impress the therapeutic conclusion of gene editing with the CRISPR/Cas9 technique [38]. Selecting an appropriate nuclease substrate and designing sgRNAs appropriately as well as certifying a suitable transition of editing approaches to purposed cells in vivo or in vitro are the first and second barriers to efficient delivery.

## 6. Methods of Delivery CRISPR/Cas System Viral Transduction

Two essential agents, Cas9 nucleases and sgRNAs, are essential for the functional activity of CRISPR/Cas9. Viral vectors are exploited to deliver genes due to the relatively high potential effect through the adhesion into the host sequences. Therefore, lentivirus vectors have been used to express Cas9 and gRNAs in organism cells and can be handy over specific sgRNAs as a knockout library in genome-scale based on CRISPR/Cas9 to screen gene activity cancer cells [39,40]. Furthermore, AAV is a nonpathogenic parvovirus that can be effective in gene delivery due to its low immunogenicity and decreased tendency of association into the host sequence and around 0.1−0.5%. The DNA packaging size of these viruses is limited to about 10 to 18 kb in length [41,42]. The CRISPR DNA entailing 1368 amino acids translated with over 4.1 kb DNA sequences is often used for a viral vector and added in multiple viral vectors that increase the processing time and cost for delivery [43].

### Non-Viral Delivery

Non-viral vectors showed a decreased immunogenicity in comparison to other vectors. Additionally, the synthesize of these vectors is simple and compatible with the synchronous transfer of sgRNAs [44]. According to the literature, there are three non-viral principles for transferring the Cas9 nucleases: the DNA containing Cas9 gene, mRNA expressing Cas9, and Cas9 protein [45]. Cas9 (CRISPR associated protein 9, formerly called Cas5, Csn1, or Csx12) is a protein that plays a vital role in the immunological defense of certain bacteria against DNA viruses and plasmids and is heavily utilized in genetic engineering applications. Its principal function is to cut DNA and thereby alter a cell’s genome.

As mentioned, Cas9 endonuclease can transfer to the host cell as mRNA. The change to the mRNA of Cas9 hampered the integration of plasmid DNA into the host genome. Additionally, delivery of Cas9 nuclease as a mRNA progenitor skelps the starting of the gene-editing process associated with CRISPR/Cas9 because it bypasses the Cas9 cDNA transcription [46,47]. Using Cas9 mRNA instead of DNA bypasses can be accomplished in the cytoplasm. In addition, the usage of Cas9 mRNA leads to instantaneous expression and eventual elimination from the body [48]. Some studies have shown that transfection of Cas9 mRNA and sgRNA by microinjection and physical delivery only causes out-of-target mutations in most cases, but microinjection is only feasible for in vitro experiments and specific approaches [38,49].

**Figure 2:**
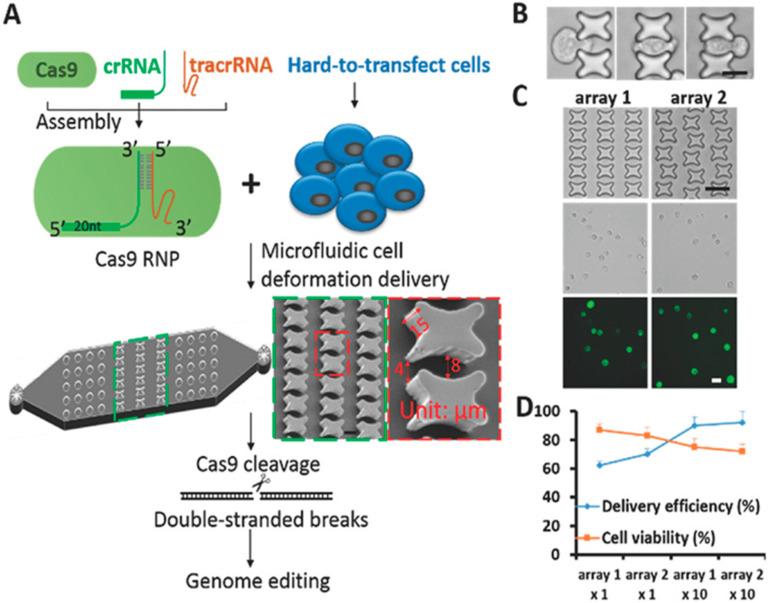
Cas9 RNP delivery strategy and chip performance. (**A**) Experimental scheme of Cas9/crRNA/tracrRNA ribonucleoprotein (Cas9 RNP) delivery for genome editing in hard-to-transfect cells via a microfluidic cell deformation chip. Scanning electron microscopy of deformable zones in the device is also shown. Scale bar, 10 µm. The red arrow indicates one curved tunnel with a depth of 15 µm and a width of 4–8 µm. (**B**) Cell deformation was shown by microscopy when SK-BR-3 cells passed through the micro construction: scale bar, 10 µm. (**C**) Microscopy of SK-BR-3 cells into which FITC-labeled 70 kDa dextran molecules were delivered through different chip designs. Arrays 1 and 2 show cell passage curved tunnels formed by different structural arrangements. Scale bar, 20 µm. (**D**) Delivery efficiency and cell viability 16 h after treatment were calculated for (**C**) at a fluid speed of 150 µL min^−1^. Array 1 × 10 or array 2 × 10 indicates cells passing through a chip with 10 repeats of identical cell deformation zones. Error bars indicate SEM (*n* = 3). (Reproduced with permission from [95] Copyright 2020 Wiley.

Although the Cas9 expression DNA is more stable and cost-effective than the Cas9 mRNA/protein, the important matter regarding the application of Cas9 expression DNA is DNA nuclear entrance [50]. The approach of plasmid DNA is harassed by accidental fusion of the plasmid DNA into the host sequence that is most severe to cells due to plasmid DNA presented to cells [51,52,53]. Additionally, cas9 protein is transferred to the mature sgRNA to manufacture a ribonucleoprotein (RNP) [54]. The complex of sgRNA does not require transcription or translation [55] and Cas9 RNPs facilitate the delivery of genomic on-target double-strand bindings after cutting by endogenous proteases [10,56]. CRISPR/Cas9 non-viral transition has been accomplished via a synthetic vector so that the process components contain the sgRNA and Cas9 nuclease encapsulates within a lipid [57], polymer, or inorganic carrier. Furthermore, this procedure was applied with the integration of the sgRNA/Cas9 nuclease into peptide sequences [58,59] (see Figure 2).

Synthetic vectors have been engineered to target specific cell populations in vivo by incorporating surface ligands onto vectors that would recognize and bind to distinct receptors on the target. These ligands can take the form of organic molecules. For example, the fusion of polyethene glycol-succinyl-Chol liposomes and folic acid molecules was undertaken by using the vector of CRISPR/Cas9 in ovarian cancer therapy. Angiopep-2, like an aptamer or protein/peptide, permits the vectors to differentiate between healthy tissues and tumor cells [60,61]. Recent non-viral methods to present Cas9/sgRNAs into cells in vitro and in vivo experiments have emphasized the physical and chemical approaches [62]. Chemical delivery exploits lipid vesicles and polymers based as vector chemicals to represent load into purposed cells; these vectors encapsulate plasmid DNA and mRNA related genetic editing [63]. Inorganic components such as gold nanoparticles, carbon nanotubes, and graphene also indicate favorable nucleic acid and protein transition [42]. Microinjection, electroporation, and cell mechanical transformation have illustrated the ability of Cas9/sgRNAs transfection [64,65]. Electrical, thermal, and mechanical forces can provide energy for physical transfection as they affect the cell membrane to permit the delivery of the loaded molecule into the cell [66]. In vivo electroporation, as a gene-editing tool, has indicated the transfection of the genetic materials into mice epidermis tissue. Likewise, a nano-needle of silicon can transfer plasmid DNA encoding the VEGF gene into the mice muscles, progressing neovascularization in the target tissue [67]. Since the physical delivery method is still too aggressive for human clinical applications applying vectors, this approach is superior for in vitro and ex vivo genome editing applications [68]. In this regard, several promising ex vivo clinical trials have been performed using engineered cells that rely on viral delivery to modify genomes. For instance, a clinical approach employing engineered T cells and through physical transfection via high-throughput ex vivo genome editing can improve cancer immunotherapy [69].

Mechano-transfection uses mechanical energies to form pores in the cell membrane through physical contact with a solid structure or a shear force from the circumambient fluids. The created pores permit specific materials to the entrance or diffuse into the cell cytoplasm. As a traditional mechanical delivery method, the microinjection method uses the buffer containing these components (genes including CRISPR or materials) injected directly through a micrometer-sized capillary into the intracellular space. Micro and nanotechnologies have utilized a mechanical portion platform to improve gene and germline editing such as single-cell applications in clinical applications [5,70,71].

## 7. Microfluidic Methods and Clinical Applications

Nowadays, one of the best approaches to deliver materials and cells is by using microfluidic channels and chips. The chips supply a suitable substrate for cell manipulation, drug screening, and characterization of the exosome. Furthermore, this device is useful for pathogen and cancer detection because of its high throughput, low cost, flexibility, and controlling fluid or gas flow [72,73]. The typical structures of a microfluidic chip like inlet, channel mixer, and outlet (or signal detector) improve gene-editing applications. Specific microfluidic devices have been fabricated using micro-/nanofabrication techniques. The new generation of microfluidics modifies nanoliter volumes of liquids or more minor and interconnected micron-sized dimension channels to provide the automated delivery of stimulatory factors to cells [74].

It is noteworthy that the manufacturing methods are different according to the material properties. The microfluidic chips manufactured in inorganic materials (silica and glass) or organic materials (polydimethylsiloxane, polystyrene, polymethyl methacrylate, or PMMA, and composite materials) [75]. Glass, silicon, and polydimethylsiloxane are the crude materials of biomedical microfluidic systems using biocompatible materials suitable for in vivo and in vitro situations and easy integration with electronics. The main biomedical analysis and finding methods are based on optical microscope technology in which the properties of silicon transparency are utilized for biomedical applications [76,77]. The liquid polydimethylsiloxane (PDMS) is prepared at high temperatures, so it is usually ripe to create soft lithographic patterns for different approaches, especially biomedical applications, tissue models, and drug testing. Some studies have shown the usage of a variety of applications and materials such as glass-based microfluidic by photolithographically etching and poly (N-isopropylacrylamide) PIPAAm with the biocompatibility of PDMS in detecting DNA samples and performing the laminar architecture of the heart ventricle [72,78,79].

Although some disadvantages of PDMS include the sorption of nonspecific and surface molecules, increased adsorption of hydrophilic molecules, the increment of incompatible solvents and reagents, and the release of uncrosslinked small PDMS molecules [80], high throughput biomedical microfluidic systems including polymethylmethacrylate (PMMA), cyclo-olefin copolymers (COC), thermoset polyester (TPE), polycarbonate (PC), polystyrene (PS), and perfluoropolyether (PFPE) are suitable for developing the microfluidic industry [81,82].

Design and production technology regarding the function of the microfluidic systems, diverse structures including arrays and multi-channels and microfluidic technologies offer an extensive range of facilities for component modification containing transportation, separation, trapping, and enrichment using hydrodynamic, acoustic, electrical, optical tweezers, and magnetic techniques. Additionally, recent micro- and nano-engineering progression offers two methods to administer particles by self-driven micro-robots in hydrogen peroxide solution and non-reciprocally beating cilia acting to deliver fluids and materials in biological systems [83,84].

Previous studies have reported that the hydrodynamic, electrical, and acoustic are the most popular tools for particle manipulation explained in CRISPR/Cas studies [85]. Additionally, microfluidics has been used for the detection of proteins (e.g., microfluidic western blotting), nucleic acids (e.g., PCR, direct detection, and DNA sequencing of nucleic acids on microfluidic chips), single cells, specific organs (organ-on-a-chip), and clinically critical small molecules [86,87,88].

The self-assembly method for fast chemical reactions and complex structures made by the injection and mixing mechanism is a new approach that depends on the channel structure for biomedical detection. For example, Y- and T-shaped multi-channels and three-dimensional junctions in the lined conduit are well-designed structures for injection [89]. In this technique, the concentration of reagent, shear force, and injection rate must be controlled [90].

To construct a 3D microfluidic system, the extracellular matrix (ECM) is the essential part of this cell-based 3D accede due to the suitable cell survival and its growth condition [72]. Therefore, the use of the ECM in the microfluidic system leads to the formation and maintenance of the binding structure of adjacent cells in vivo to provide a platform for imitation of the real 3D microenvironment for medical models [91]. The ECM in the microfluidic approach provides pivotal functions including supplying an ideal microenvironment in the system for cell growth, differentiating cell to vital organs, and manufacturing high potency models for cell aggregation, spheroid, tissue or organ models [92].

The examinations in 3D HTBMS were performed on a varied range of applications such as 3D cell culture based on microfluidics and the organ-on-a-chip approach [93]. Zhu et al. (2019) showed a novel 3D micro-assembling strategy assisted by 3D printing, enabling the molding of 3D microstructures such as LEGO^®^ parts from 3D-printed molds. In this study, the molded PDMS LEGO assembled into a 3D-cell culture chamber interconnected with vertical and horizontal perfusion microchannels such as a 3D channel network by multi-directional electric frequency scanning (3D μ-electro-transfection). This generates local fluctuation to enhance mass transport via the interconnected vertical and horizontal perfusion microchannel array, which was reconstructed by this 3D printing-assisted molding and assembling process, thus improving cell transfection efficiency [94].

Molecular techniques such as CRISPR-based diagnosis may provide new responses to molecular diagnosis. Perhaps the ability to purposefully edit the genome is crucial to understanding the genetic contribution to biology and disease. On the other hand, among the different cell sources, T cell genome editing holds good promise for immunotherapy for cancer, human immunodeficiency virus (HIV), primary immunodeficiency, and autoimmune diseases, but genetically engineered high-yield human T cells are challenging [10]. The CRISPR/Cas9 technique can be extended using the bacteria’s immune system in which these spacer sequences from viruses and phages integrate into the prokaryotes including bacteria. However, the CRISPR-Cas9 technology facilitates genome editing in many cell types, and its efficiency has been limited in difficult-to-transfect cells such as primary human T cells (Table 1) [96].

In a high throughput study, Han et al. (2019) fabricated a delivery chip and enhanced the transition efficiency of Cas9 RNPs into different cell types including human primary CD4+ T cells as well as facilitate Cas9 RNP-directed genome editing. The improved Cas9/sgRNA ribonucleoprotein (RNP) complex transmission enabled a decrease in the off-target effects, preventing unpleasant plasmid sequence entry. In addition, knock-in genome amendment in primary T cells was achieved in PD-1 as a programmed cell death protein because creating the genetic deletion in PD-1 was proven advantageous in engineering T cells for cancer and tumor immunotherapies. According to this study, microfluidic cell deformation-based Cas9 RNP delivery provided accurate genome editing in T cells and cancer immunotherapy [95].

In another study, Han et al. (2016) reported a loss of CRISPR-Cas9-mediated kinase function for cancer cell deformation and invasive potential in a properly functioning microfluidic chip. They combined the CRISPR-Cas9 screen with chip-based cell deformation sorting to identify tumor-inhibiting kinases and designed a unique cell purification system to sort highly deformable cells in a high-efficiency method. This microfluidic device allows flexible cells with high ductility and metastatic tendency to cross micro-barriers and exit the separation chip under hydrodynamic forces, while rigid cells remain trapped [52]. Initially, microfluidic deformable chip cell separation or MS-chip (mechanical separation chip) was designed and validated to separate flexible cells from rigid cells by hydrodynamic forces (separating structure consists of two million rectangular micro-posts 30 mm in height with gap distances decreasing from 15 mm to 6 mm). Their study provided a new perspective for the large-scale coalition of the lab-on-chip example to rapidly screen gene function based on the CRISPR knockout system [97].

Because cancer is a heterogeneous disease that is genetically very different and relies on different pathways for survival, chemical susceptibility testing shifts to a more personal approach. As expected, one of the preferred methods is to use the CRISPR approach to screen and recognize genes involved in cell proliferation in mammalian models [98]. Sinha et al. first showed automatic gene editing through digital microfluidics by a system to decode the genes related to lung cancer in transfer and deletion efficiency with a standard imaging pipeline. Additionally, gene-editing evaluation in this study targeted edit automation of the MAPK/ERK pathway, especially the RAF1 gene, to show the function of digital microfluidics in culturing lung cancer cells for a long time and implement gene transfection and knockout procedures for cancer therapy [74,99].

Among the technologies that have been realized for the delivery of exogenous molecules into living cells in vivo or in vitro experiments, electroporation has successfully been recognized as one of the most varied and flexible approaches with the most transfection ability and minimum cell toxicity. mRNA delivery can be demonstrated efficiently into mouse zygotes for CRISPR/Cas9-based genome editing and used to extract genomic DNA or release other intracellular components from cells through microfluidic systems [100]. Duo to the shortage of the high throughput tool in genetic screenings, cellular analysis, and delivery, selective release of intracellular molecules at the single-cell level and detection of kinase translocation several groups including Geng et al. [101], Bao et al. [102], and Wang et al. [103], respectively, have improved the diversity of minimized electroporation devices by obtaining the advantages of micromachining.

In this regard, Bian et al. developed a high-throughput electroporation microsystem with an optimized superhydrophobic feature. They used the micro-well array to attain controlled conditions in each micro-well and the digital electrode in micro-wells to increase efficient transfection and electroporation of ingredients such as sgRNA into the cells. Two plasmids encoding enhanced green and red fluorescent proteins were successfully delivered into HeLa cells on a 169-microwell array chip. Additionally, the sgRNA was conducted through selective electroporation into 293T cells expressing the Cas9 nuclease [104]. Previously, Bian’s group developed a superhydrophobic microwell array chip (SMARchip) for enhanced screening in cell culture. The SMARchip platform performed the successful combining of stem cells with chemical and mechanical elements. During electroporation, each electrode unit made contact with the corresponding microwells through droplet connections and the sgRNAs within cells were thoroughly examined in this approach [105].

DiTommaso et al. used a genome-wide approach to study optimized electroporation treatment and recognize significant disruptions in the expression profiles in transcripts of human T cells. They found that a microfluidic membrane deformation technique denominated “squeezing” had lesser side effects than electroporation. They determined changes at the genetic level with full transcriptome microarrays, accredited protein expression, and specific phenotype through some markers. In the direction of comparing the electroporation and cell squeezing, they selected two cell types that are common targets for gene engineering: CD4+ HSCs and T cells. Delivery of fluorescent-tagged dextran to cells isolated from human donors via electroporation or cell-squeezing protocols was performed, and Cas9 protein–gRNA RNP complexes (Cas9 RNPs) were transfected to target PD-1 via gene-editing of both technologies. As mentioned in previous studies, Cas9 RNPs process an arresting system for manipulating the genomes of primary cells, especially T cells [106,107]. To evaluate the function of this system, they selected two critically expressed genes of T cell, IFN γ, and IL-2 to characterize their expression levels after treatment with the delivery process. As a result, cells targeted to a mechanical membrane disruption-based delivery mechanism or cell squeezing had minimal aberrant transcriptional responses, and T cells edited with similar efficiency via cell squeezing and microfluidic approach compared to electroporation demonstrated the expected tumor-killing advantage.

Their work suggests that mechanical membrane disconnection coupled with diffusion-mediated delivery significantly decreases unintended negative consequences and functional deficiencies. The significant differences in outcomes from the two techniques emphasized the importance of intracellular transfection methods on cell function research and clinical applications [108].

Cas9 was extracted from many species of bacteria and applied in biotechnology, so the capability of Cas9 detecting in *S. pyogenes*, *S. aureus*, *N. meningitides*, and *S. thermophiles* was developed using approved antibodies. The use of Cas9 varied species due to having specific properties in their genomes improved structures with thermal stability and smaller size [43,109,110]. Phaneuf C et al. exerted a centrifugal microfluidic platform, multiple Cas9 species, and a dCas9 (dead Cas9) integrated into effector domains to detect both Cas9 protein levels and the function of catalytic nuclease.

The Dcas9 scaffold is composed of two components including the nuclease domains inactivated by single-base mutation and the Cas9 attached guide-RNA remaining in the DNA sequences. Targeted mutations have been created by integrating a cytidine deaminase domain to dCas9 [25]. Microfluidic discs were made from polymethyl methacrylate (PMMA) and PSA film as a pressure-sensitive adhesive. Indeed, the laser beams cut sections of PMMA and PSA along with alignment holes that allow for gathering efficiently via spired pins. The layers were then placed against each other, and the disks were placed in a vacuum for 24 h before being used. Each of the three centrifugal microfluidic layers constructed with CO_2_ laser and two ends of the structure was composed of a rigid PMMA layered assembly with an adhesive layer.

To detect Cas9 species, the silica microparticles combined with the CRISPR inhibitor AcrIIC1 protein binds to the conserved HNH domain from many species of Cas9 [111]. Because this, a protein accompanied by a specific antibody detector could identify the Cas9 in species such as N. meningitides, G. stearothermophilus, and C. jejuni [112,113]. They have shown dramatic enhancement through a manual microfluidic tool to detect fluorescence signals and trace Cas9 multiple species directly in the bodily fluids that are useful in preclinical experiments [25]. CRISPR-based diagnostic methods have aroused a high enthusiasm due to their efficiency, sensitivity, and high proprietary [114].

Brush et al. integrated the first clustered regularly interspaced short palindromic repeats (CRISPR)/Cas13a-powered microfluidics with an electrochemical signal readout to identify microRNAs, increase sensitivity as a target amplification, and selective diagnostic test in another publication. However, there is an urgent need to develop handmade methods for miRNA detection in clinical trials, where the quantification of the potential tumor marker microRNAs can be recognized without amplifying genomic sequences. They combined the CRISPR technology with an electrochemical microfluidic equipped with the DFR technology (Dry Film photo-Resist) as a biosensor to evaluate miRNA levels of the brain tumor marker miR-19b in serum samples of cancer patients. The specificity of the detection is given by a specific CRISPR RNA (crRNA) that guides the Cas13a to the target sequence of RNA [115]. After recognizing the complementary RNA sequence, the cleavage ability of the enzyme is activated, and a reporter RNA can be used for a quantitative assessment of RNA levels. Arranging multiple DFR foils into a platinum patterned polyimide substrate, the electrodes, and the microchannels consisting of an electrochemical cell, was realized to operate their functions.

As a result, this unique merger of the CRISPR/Cas13a technology with the microfluidic biosensor equipped with an electrochemical reader, permits the detection of the target miRNAs including miR-19b and miR-20a with a high sensitivity method [36].

Rapid and accurate adjustable molecular diagnostic tests such as CRISPR-based approaches are imperative to stop the global spread of new diseases. The spread of COVID-19 throughout the world has disclosed significant notches in clinical to react against new viral pathogens. In one study by Ramachandran et al., the CRISPR-Cas12 system complexed with a synthetic guide RNA, and this structure is activated when it highly binds explicitly to target DNA and randomly cleaves single-stranded DNA through probes labelled with a fluorophore-quencher pair. Then, they achieved an appropriate electric field gradient using a particular ionic focusing technique such as an isotachophoresis method on a microfluidic chip. They also combined isotachophoresis with loop-mediated isothermal amplification to detect SARS-CoV-2 RNA in 30 min that provides automated purification of target RNA from a raw nasopharyngeal swab sample and subsequently diagnose SARS-CoV-2 RNA based on microfluidic-CRISPR assays. Isotachophoresis or ITP is an electro-kinetic microfluidic technique that uses a two-buffer system including a high-mobility leading electrolyte and a low-mobility trailing electrolyte buffer (the combination of microfluidics and on-chip electric field). This combination used the desired on-chip ITP to effect rapid CRISPR-Cas12 enzymatic activity and focused Cas12-gRNA upon target nucleic acid recognition. Briefly, a novel electric field has improved the microfluidic method, which is enforceable for the field of CRISPR diagnostics [116,117,118].

**Table 1:** The clinical application of employing microfluidic methods for gene delivery.

Target	Transfection Method	Experiments	Results	Ref.
Cas9/RNPs	Microfluidic Rapid mechanical deformation to shear cells for delivery	The fabrication of the delivery chip and enhanced the transition efficiency of Cas9 RNPs into human primary CD4+ T cells	Knock-in genome amendment in primary T cells PD-1 as an engineering T cells for cancer and tumor immunotherapies	Han, X. et al., 2017 [95]
CRISPR/Cas9	Microfluidic Rapid mechanical deformation of cells to shear cells	The combination of CRISPR-Cas9 screen with chip-based cell deformation sorting highly deformable cells in a high-efficiency method	Identify tumor-inhibiting kinases and loss of CRISPR-Cas9-mediated kinase function for cancer cell deformation and invasive potential	Han, X. et al., 2016 [97]
CRISPR/Cas9	Microfluidic Rapid mechanical deformation of cells to shear cells	Optimizing the physical constriction in a microfluidic setup for delivering ssDNA, siRNAs, and large-sized plasmids into different cell types, adherent and non-adherent cells	Approaching high delivery efficiency of macromolecules into hard-to-transfect lymphoma cells and embryonic stem cells, with maintaining high cell viability	Han, X. et al., 2015 [52]
CRISPR/Cas9	Microfluidic A new droplet-based method along with chemical delivery through lipid vesicles	Digital microfluidics (DMF) for targeting to edit automated the MAPK/ERK pathway, especially RAF1 gene with and without a Raf-1 inhibitor	Decoding the genes related to lung cancer in terms of transfer and deletion efficiency with a standard imaging pipeline	Sinha, H. et al., 2018 [74]
CRISPR/Cas9	Microfluidic A high-throughput electroporation microsystem (electrical force)	Two plasmids encoding green and red fluorescent proteins electroporated into HeLa cells on a 169-microwell array chip	Conducting sgRNA into 293T cells that are expressing the Cas9 nuclease for increasing efficient transfection	Bian, S. et al., 2017 [104]
CRISPR/Cas9	Electroporation treatment & mechanical membrane disruption-based microfluidic	Comparing electroporation with microfluidic membrane deformation technique or “cell squeezing” with in two cell types: HSC & T cells to target PD-1	Squeezing had minimal aberrant transcriptional responses and T cells edited had a tumor-killing function	Ditommaso, T. et al., 2018 [108]
CRISPR/Cas9	Microfluidic integrated with chemical delivery and inducible CRISPR-Cas construct for detection yeast	Evaluation DNA damage or genomic instability behave in induction with genotoxic chemicals (MMS, HU) or a single double-strand break using induced CRISPR-Cas9 expression	A device for long-term imaging of yeast cells under stable or varied media conditions Permitting acquisition of high-resolution images from cells fixing in a two dimensional imaging plane	Schmidt, G.W. et al., 2017 [119]
CRISPR/Cas9	A centrifugal microfluidic platform with fluorescence-based sedimentation nuclease assay for detection	Multiple Cas9 species and a dCas9 (dead Cas9) integrated into effector domains for detecting of both Cas9 protein levels and function of catalytic nuclease	Improving a manual microfluidic tool for the detection of fluorescence signals and tracing of Cas9 multiple species directly in the bodily fluids	Phaneuf, C. et al., 2019 [25]
CRISPR/Cas13a	Microfluidic integrated electrochemical biosensor for the on-site detection	Combining the CRISPR technology with a microfluidic equipped with the DFR technology (dry film photo-resist) to evaluate miRNA levels	Permitting the detection of the target miRNAs, including miR-19b and miR-20a as brain tumor markers with high sensitivity method	Brush, R. et al., 2019 [36]
CRISPR/Cas13a	Microfluidic integrated a photocleavable capture probe and a sequence-specific barcode fluorescence reporter	Mixing automated Microfluidic, hybridization and measuring nonspecific cleavage products of Cas13a by a custom integrated fluorometer in small size and in-field diagnosis	Applying an automated point of care system for Ebola RNA detection with RNA-guided RNA endonuclease Cas13a, Achieving within 5 min, a detection limit of 20 pfu/mL of purified Ebola RNA	Qin, P. et al., 2019 [115]
CRISPR/Cas12	Microfluidic An integrated micropillar polydimethylsiloxane Microinjection for viral detection	After modification surface, the reporter probes were firstly immobilized in the enclosed channel, and the CRISPR Cas12a/crRNA complex was injected into the fully enclosed system for non-specifically cleaves the ssDNA reporters	Using the high activity of the CRISPR-Cas12a enzyme and the ability of micropillars (IMPACT chip) to bind more reporter probe or pathogens and the next-generation molecular diagnostic platform for point-of-care applications	Hass, K.N. et al., 2020 [120]
CRISPR/Cas12	Microfluidic integrated electric field gradient using a selective ionic focusing detection (isotachophoresis)	Combining CRISPR-Cas12 system with an electro-kinetic microfluidic technique and loop-mediated isothermal amplification to evaluate viruses	Diagnosing SARS-CoV-2 in 30 min that provide automated purification of target RNA from a nasopharyngeal swab sample	Ramachandran, A. et al., 2020 [118]

## 8. Conclusions

The CRISPR/Cas approaches suggest advantages in gene-editing technology through a guide RNA and CAS nuclease to recognize and manipulate the proposed DNA genome by respecting efficiency and specificity. This versatile gene-editing system has progressed swiftly, and various utilizations from disease diagnosis to therapeutic intermediations have been supported through this method. However, transferring is the main subject for accomplishing CRISPR/Cas gene editing in clinical experiments. A well-designed CRISPR/Cas survey should consider the different aspects including attaining maximum on-target efficiency, reducing off-target effects, and accurate transferring. In the past decade, microfluidic technologies have been widely implemented in many fields ranging from fundamental biological research and targeted delivery via microfluidic chips would enhance the use of this technology. The microfluidic device has great potential for complex sample processing and a high level of integration, making them robust and powerful tools in point-of-care testing. Results show that the combined microfluidic sorting systems based on the mechanical properties of cells with CRISPR-Cas9 technologies is a potential approach for gene editing, clinical therapy, and a genetic screening strategy that facilitates the rapid identification of gene function in abundant diseases. To summarize, this genome editing application has been continued to apply novelties and vast opportunities with close collaboration between biomedical and biomaterial researchers who can create communication via improving impressive non-viral physical transfection methods such as a microfluidics method for various gene-editing and therapy factors.

## Data Availability

Not applicable.

## References

[1] Han H.A., Pang J.K.S., Soh B.-S. (2020). Mitigating off-target effects in CRISPR/Cas9-mediated in vivo gene editing. J. Mol. Med..

[2] Blaese R.M., Culver K.W., Miller A.D., Carter C.S., Fleisher T., Clerici M., Shearer G., Chang L., Chiang Y., Tolstoshev P. (1995). T Lymphocyte-Directed Gene Therapy for ADA- SCID: Initial Trial Results After 4 Years. Science.

[3] Ho B.X., Loh S.J.H., Chan W.K., Soh B.S. (2018). In Vivo Genome Editing as a Therapeutic Approach. Int. J. Mol. Sci..

[4] Gaj T., Gersbach C.A., Barbas C.F. (2013). ZFN, TALEN, and CRISPR/Cas-based methods for genome engineering. Trends Biotechnol..

[5] Horii T., Arai Y., Yamazaki M., Morita S., Kimura M., Itoh M., Abe Y., Hatada I. (2014). Validation of microinjection methods for generating knockout mice by CRISPR/Cas-mediated genome engineering. Sci. Rep..

[6] Deng D., Yan C., Pan X., Mahfouz M., Wang J., Zhu J.-K., Shi Y., Yan N. (2012). Structural Basis for Sequence-Specific Recognition of DNA by TAL Effectors. Science.

[7] Yang Y., Xu J., Ge S., Lai L. (2021). CRISPR/Cas: Advances, Limitations, and Applications for Precision Cancer Research. Front. Med..

[8] Cyranoski D. (2016). Chinese scientists to pioneer first human CRISPR trial. Nat. Cell Biol..

[9] Li Y., Glass Z., Huang M., Chen Z.-Y., Xu Q. (2020). Ex vivo cell-based CRISPR/Cas9 genome editing for therapeutic applications. Biomaterials.

[10] Li L., He Z.-Y., Wei X.-W., Gao G.-P., Wei Y.-Q. (2015). Challenges in CRISPR/CAS9 delivery: Potential roles of non-viral vectors. Hum. Gene Ther..

[11] Elvira K.S. (2021). Microfluidic technologies for drug discovery and development: Friend or foe?. Trends Pharmacol. Sci..

[12] Chiu D.T., Demello A.J., Di Carlo D., Doyle P.S., Hansen C., Maceiczyk R.M., Wootton R.C. (2017). Small but Perfectly Formed? Successes, Challenges, and Opportunities for Microfluidics in the Chemical and Biological Sciences. Chem.

[13] Shepherd S.J., Issadore D., Mitchell M.J. (2021). Microfluidic formulation of nanoparticles for biomedical applications. Biomaterials.

[14] Yixiao L., Jianzhang P.A.N., Qun F. (2021). Research advances of high-throughput cell-based drug screening systems based on microfluidic technique. Chin. J. Chromatogr..

[15] Levy S.L., Craighead H.G. (2010). DNA manipulation, sorting, and mapping in nanofluidic systems. Chem. Soc. Rev..

[16] Ottino J.M., Wiggins S. (2004). Introduction: Mixing in microfluidics. Philos. Trans. R. Soc. A Math. Phys. Eng. Sci..

[17] Balbino T.A., Azzoni A.R., De La Torre L.G. (2013). Microfluidic devices for continuous production of pDNA/cationic liposome complexes for gene delivery and vaccine therapy. Colloids Surf. B Biointerfaces.

[18] Laohakunakorn N., Lavickova B., Swank Z., Laurent J., Maerkl S.J. (2021). Steady-state cell-free gene expression with microfluidic chemostats. Synthetic Gene Circuits.

[19] Ahmadi S., Rabiee N., Bagherzadeh M., Karimi M. (2021). Microfluidic devices for gene delivery systems. Biomed. Appl. Microfluid. Devices.

[20] Kim J., Hwang I., Britain D., Chung T.D., Sun Y., Kim D.-H. (2011). Microfluidic approaches for gene delivery and gene therapy. Lab. Chip.

[21] Niculescu A.-G., Chircov C., Bîrcă A., Grumezescu A. (2021). Fabrication and Applications of Microfluidic Devices: A Review. Int. J. Mol. Sci..

[22] Rautela I., Uniyal P., Thapliyal P., Chauhan N., Sinha V.B., Sharma M.D. (2021). An extensive review to facilitate under-standing of CRISPR technology as a gene editing possibility for enhanced therapeutic applications. Gene.

[23] Zhang S., Shen J., Li D., Cheng Y. (2021). Strategies in the delivery of Cas9 ribonucleoprotein for CRISPR/Cas9 genome editing. Theranostics.

[24] Leibowitz M.L., Papathanasiou S., Doerfler P.A., Blaine L.J., Sun L., Yao Y., Zhang C.-Z., Weiss M.J., Pellman D. (2021). Chromothripsis as an on-target consequence of CRISPR–Cas9 genome editing. Nat. Genet..

[25] Phaneuf C.R., Seamon K.J., Eckles T.P., Sinha A., Schoeniger J.S., Harmon B., Meagher R.J., Abhyankar V.V., Koh C.-Y. (2019). Ultrasensitive multi-species de-tection of CRISPR-Cas9 by a portable centrifugal microfluidic platform. Anal. Methods.

[26] Makarova K.S., Wolf Y.I., Iranzo J., Shmakov S.A., Alkhnbashi O.S., Brouns S.J.J., Charpentier E., Cheng D., Haft D.H., Horvath P. (2019). Evolutionary classification of CRISPR–Cas systems: A burst of class 2 and derived variants. Nat. Rev. Microbiol..

[27] Yang H., Ren S., Yu S., Pan H., Li T., Ge S., Zhang J., Xia N. (2020). Methods Favoring Homology-Directed Repair Choice in Response to CRISPR/Cas9 Induced-Double Strand Breaks. Int. J. Mol. Sci..

[28] Doudna J.A., Sontheimer E.J. (2014). The Use of CRISPR/cas9, ZFNs, TALENs in Generating Site-Specific Genome Alterations.

[29] Qasim W., Zhan H., Samarasinghe S., Adams S., Amrolia P., Stafford S., Butler K., Rivat C., Wright G., Somana K. (2017). Molecular remission of infant B-ALL after infusion of universal TALEN gene-edited CAR T cells. Sci. Transl. Med..

[30] Zhang B. (2021). CRISPR/Cas gene therapy. J. Cell. Physiol..

[31] Abudayyeh O.O., Gootenberg J.S., Konermann S., Joung J., Slaymaker I.M., Cox D.B.T., Shmakov S., Makarova K.S., Semenova E., Minakhin L. (2016). C2c2 is a single-component programmable RNA-guided RNA-targeting CRISPR effector. Science.

[32] Rusk N. (2019). Spotlight on Cas12. Nat. Chem. Biol..

[33] East-Seletsky A., O’Connell M.R., Knight S.C., Burstein D., Cate J.H.D., Tjian R., Doudna J.A. (2016). Two distinct RNase activities of CRISPR-C2c2 enable guide-RNA processing and RNA detection. Nat. Cell Biol..

[34] Watanabe S., Cui B., Kiga K., Aiba Y., Tan X.-E., Sato’O Y., Kawauchi M., Boonsiri T., Thitiananpakorn K., Taki Y. (2019). Composition and Diversity of CRISPR-Cas13a Systems in the Genus Leptotrichia. Front. Microbiol..

[35] Freije C.A., Myhrvold C., Boehm C.K., Lin A.E., Welch N.L., Carter A., Metsky H.C., Luo C., Abudayyeh O.O., Gootenberg J.S. (2019). Programmable inhibition and detection of RNA viruses using Cas13. Mol. Cell.

[36] Bruch R., Baaske J., Chatelle C., Meirich M., Madlener S., Weber W., Dincer C., Urban G. (2019). CRISPR/Cas13a-powered electro-chemical microfluidic biosensor for nucleic acid amplification-free miRNA diagnostics. Adv Mater..

[37] Gootenberg J.S., Abudayyeh O.O., Lee J.W., Essletzbichler P., Dy A.J., Joung J., Verdine V., Donghia N., Daringer N.M., Freije C.A. (2017). Nucleic acid detection with CRISPR-Cas13a/C2c2. Science.

[38] Wang H.-X., Li M., Lee C.M., Chakraborty S., Kim H.-W., Bao G., Leong K.W. (2017). CRISPR/Cas9-based genome editing for disease modeling and therapy: Challenges and opportunities for non-viral delivery. Chem Rev..

[39] Kumar M., Keller B., Makalou N., Sutton R.E. (2001). Systematic Determination of the Packaging Limit of Lentiviral Vectors. Hum. Gene Ther..

[40] Chen X., Gonçalves M.A.F.V. (2016). Engineered viruses as genome editing devices. Mol. Ther..

[41] Esvelt K.M., Mali P., Braff J.L., Moosburner M., Yaung S.J., Church G.M. (2013). Orthogonal Cas9 proteins for RNA-guided gene regulation and editing. Nat. Methods.

[42] Fajrial A.K., He Q.Q., Wirusanti N.I., Slansky J.E., Ding X. (2020). A review of emerging physical transfection methods for CRISPR/Cas9-mediated gene editing. Theranostics.

[43] Ran F.A., Cong L., Yan W.X., Scott D.A., Gootenberg J.S., Kriz A.J., Zetsche B., Shalem O., Wu X., Makarova K.S. (2015). In vivo genome editing using Staphylococcus aureus Cas9. Nature.

[44] Lin C.-Y., Hsieh H.-Y., Pitt W.G., Huang C.-Y., Tseng I.-C., Yeh C.-K., Wei K.-C., Liu H.-L. (2015). Focused ultrasound-induced blood-brain barrier opening for non-viral, non-invasive, and targeted gene delivery. J. Control. Release.

[45] Li S.-D., Huang L. (2007). Non-viral is superior to viral gene delivery. J. Control. Release.

[46] Kim S., Kim D., Cho S.W., Kim J., Kim J.-S. (2014). Highly efficient RNA-guided genome editing in human cells via de-livery of purified Cas9 ribonucleoproteins. Genome Res..

[47] Glass Z., Lee M., Li Y., Xu Q. (2018). Engineering the Delivery System for CRISPR-Based Genome Editing. Trends Biotechnol..

[48] Miller J.B., Zhang S., Kos P., Xiong H., Zhou K., Perelman S.S., Zhu H., Siegwart D.J. (2017). Non-Viral CRISPR/Cas Gene Editing In Vitro and In Vivo Enabled by Synthetic Nanoparticle Co-Delivery of Cas9 mRNA and sgRNA. Angew Chem. Int. Ed..

[49] Mali P., Yang L., Esvelt K.M., Aach J., Guell M., DiCarlo J., Norville J.E., Church G.M. (2013). RNA-Guided Human Genome Engineering via Cas9. Science.

[50] Sakuma T., Nishikawa A., Kume S., Chayama K., Yamamoto T. (2015). Multiplex genome engineering in human cells using all-in-one CRISPR/Cas9 vector system. Sci. Rep..

[51] Cong L., Ran F.A., Cox D., Lin S., Barretto R., Habib N., Hsu P.D., Wu X., Jiang W., Marraffini L.A. (2013). Multiplex Genome Engineering Using CRISPR/Cas Systems. Science.

[52] Han X., Liu Z., Jo M.C., Zhang K., Li Y., Zeng Z., Li N., Zu Y., Qin L. (2015). CRISPR-Cas9 delivery to hard-to-transfect cells via membrane deformation. Sci. Adv..

[53] Perez-Pinera P., Kocak D.D., Vockley C.M., Adler A.F., Kabadi A.M., Polstein L.R., Thakore P.I., Glass K.A., Ousterout D.G., Leong K.W. (2013). RNA-guided gene activa-tion by CRISPR-Cas9–based transcription factors. Nat. Methods.

[54] Mout R., Ray M., Tonga G.Y., Lee Y.-W., Tay T., Sasaki K., Rotello V.M. (2017). Direct Cytosolic Delivery of CRISPR/Cas9-Ribonucleoprotein for Efficient Gene Editing. ACS Nano..

[55] Chew W.L., Tabebordbar M., Cheng J.K.W., Mali P., Wu E.Y., Ng A.H.M., Zhu K., Wagers A.J., Church G.M. (2016). A multifunctional AAV–CRISPR–Cas9 and its host response. Nat. Methods.

[56] Wang M., Zuris J.A., Meng F., Rees H., Sun S., Deng P., Han Y., Gao X., Pouli D., Wu Q. (2016). Efficient delivery of genome-editing proteins using bioreducible lipid nanoparticles. Proc. Natl. Acad. Sci. USA.

[57] Zuris J.A., Thompson D.B., Shu Y., Guilinger J.P., Bessen J.L., Hu J.H., Maeder M.L., Joung K.J., Chen Z.-Y., Liu D.R. (2015). Cationic lipid-mediated delivery of proteins enables efficient protein-based genome editing in vitro and in vivo. Nat. Biotechnol..

[58] Kauffman K.J., Dorkin J.R., Yang J.H., Heartlein M.W., DeRosa F., Mir F.F., Fenton O.S., Anderson D.G. (2015). Optimization of lipid nanoparticle formulations for mRNA delivery in vivo with fractional factorial and definitive screening designs. Nano. Lett..

[59] Ginn S.L., Amaya A.K., Alexander I.E., Edelstein M., Abedi M.R. (2018). Gene therapy clinical trials worldwide to 2017: An update. J. Gene Med..

[60] Li L., Wang H., Ong Z.Y., Xu K., Ee P.L.R., Zheng S., Hedrick J.L., Yang Y.-Y. (2010). Polymer- and lipid-based nanoparticle therapeutics for the treatment of liver diseases. Nano. Today.

[61] Steichen S.D., Caldorera-Moore M., Peppas N.A. (2013). A review of current nanoparticle and targeting moieties for the delivery of cancer therapeutics. Eur. J. Pharm. Sci..

[62] Rahimi H., Salehiabar M., Charmi J., Barsbay M., Ghaffarlou M., Razlighi M.R., Davaran S., Khalilov R., Sugiyama M., Nosrati H. (2020). Harnessing nanoparticles for the efficient delivery of the CRISPR/Cas9 system. Nano. Today.

[63] Yu X., Liang X., Xie H., Kumar S., Ravinder N., Potter J., de Mollerat du Jeu X., Chesnut J.D. (2016). Improved delivery of Cas9 protein/gRNA com-plexes using lipofectamine CRISPRMAX. Biotechnol Lett..

[64] Stewart M.P., Langer R., Jensen K.F. (2018). Intracellular Delivery by Membrane Disruption: Mechanisms, Strategies, and Concepts. Chem. Rev..

[65] O’Dea S., Annibaldi V., Gallagher L., Mulholland J., Molloy E.L., Breen C.J., Gilbert D.S.M., Maguire M., Curry F.-R. (2017). Vector-free intracellular delivery by reversible permeabilization. PLoS ONE.

[66] Meacham J.M., Durvasula K., Degertekin F.L., Fedorov A.G. (2014). Physical methods for intracellular delivery: Practical aspects from laboratory use to industrial-scale processing. J. Lab. Autom..

[67] Chiappini C., De Rosa E., Martinez O.J., Liu X., Steele J.R., Stevens M.M., Tasciotti E. (2015). Biodegradable silicon nanoneedles delivering nucleic acids intracellularly induce localized in vivo neovascularization. Nat. Mater..

[68] Fajrial A.K., Ding X. (2019). Advanced nanostructures for cell membrane poration. Nanotechnology.

[69] Stadtmauer E.A., Fraietta J.A., Davis M.M., Cohen A.D., Weber K.L., Lancaster E., Mangan P.A., Kulikovskaya I., Gupta M., Chen F. (2020). CRISPR-engineered T cells in patients with refractory cancer. Science.

[70] Hruscha A., Schmid B. (2015). Generation of zebrafish models by CRISPR/Cas9 genome editing. Neuronal Cell Death.

[71] Crispo M., Mulet A.P., Tesson L., Barrera N., Cuadro F., dos Santos-Neto P.C., Nguyen T.H., Creneguy A., Brusselle L., Anegon I. (2015). Efficient generation of myo-statin knockout sheep using CRISPR/Cas9 technology and microinjection into zygotes. PLoS ONE.

[72] Song K., Li G., Zu X., Du Z., Liu L., Hu Z. (2020). The Fabrication and Application Mechanism of Microfluidic Systems for High Throughput Biomedical Screening: A Review. Micromachines.

[73] Yang Y., Chen Y., Tang H., Zong N., Jiang X. (2020). Microfluidics for Biomedical Analysis. Small Methods.

[74] Sinha H., Quach A.B.V., Vo P.Q.N., Shih S.C.C. (2018). An automated microfluidic gene-editing platform for deciphering cancer genes. Lab. Chip.

[75] Kelly R.T., Lin J.M. (2018). Cell analysis on microfluidics. Anal. Bioanal. Chem..

[76] Oshchepkov M.S., Kovalenko L.V., Kotova Y.O., Solov’Eva I.N., Bystrova N.A., Kochetkov K.A. (2020). Potential of Application of Microfluidic Devices in Preparative Chemistry. INEOS OPEN.

[77] Silverio V., de Freitas S.C. (2018). Microfabrication techniques for microfluidic devices. Complex Fluid-Flows in Microfluidics.

[78] Han J.P., Sun J., Wang L., Liu P., Zhuang B., Zhao L., Liu Y., Li C.X. (2017). The optimization of electrophoresis on a glass micro-fluidic chip and its application in forensic science. J. Forensic Sci..

[79] Agarwal A., Goss J.A., Cho A., McCain M.L., Parker K.K. (2013). Microfluidic heart on a chip for higher throughput pharmacological studies. Lab. Chip.

[80] Alrifaiy A., Lindahl O.A., Ramser K. (2012). Polymer-Based Microfluidic Devices for Pharmacy, Biology and Tissue Engineering. Polymers.

[81] Liu Y., Hu K., Wang Y. (2017). Primary Hepatocytes Cultured on a Fiber-Embedded PDMS Chip to Study Drug Metabolism. Polymers.

[82] Oderinde O., Liu S., Li K., Kang M., Imtiaz H., Yao F., Fu G. (2018). Multifaceted polymeric materials in three-dimensional processing (3DP) technologies: Current progress and prospects. Polym. Adv. Technol..

[83] Lu X., Liu C., Hu G., Xuan X. (2017). Particle manipulations in non-Newtonian microfluidics: A review. J. Colloid Interface Sci..

[84] Kim K., Guo J., Liang Z., Fan D. (2018). Artificial Micro/Nanomachines for Bioapplications: Biochemical Delivery and Diagnostic Sensing. Adv. Funct. Mater..

[85] Zhang S., Wang Y., Onck P., Toonder J.D. (2020). A concise review of microfluidic particle manipulation methods. Microfluid. Nanofluidics.

[86] Chang H.-N., Leroueil P.R., Selwa K., Gasper C.J., Tsuchida R.E., Wang J.J., McHugh W.M., Cornell T.T., Baker J.R., Goonewardena S.N. (2013). Profiling Inflammatory Responses with Microfluidic Immunoblotting. PLoS ONE.

[87] Shen H., Qu F., Xia Y., Jiang X. (2018). Straightforward and Ultrastable Surface Modification of Microfluidic Chips with Norepinephrine Bitartrate Improves Performance in Immunoassays. Anal. Chem..

[88] Ma J., Lee S.M.-Y., Yi C., Li C.-W. (2017). Controllable synthesis of functional nanoparticles by microfluidic platforms for biomedical applications–A review. Lab. Chip.

[89] Ran R., Middelberg A.P., Zhao C.-X. (2016). Microfluidic synthesis of multifunctional liposomes for tumour targeting. Colloids Surf. B Biointerfaces.

[90] Yang M., Zhang W., Zheng W., Cao F., Jiang X. (2017). Inkjet-printed barcodes for a rapid and multiplexed paper-based assay compatible with mobile devices. Lab. Chip.

[91] Hinderer S., Layland S.L., Schenke-Layland K. (2016). ECM and ECM-like materials—Biomaterials for applications in regenerative medicine and cancer therapy. Adv. Drug Deliv. Rev..

[92] Domachuk P., Tsioris K., Omenetto F.G., Kaplan D.L. (2009). Bio-microfluidics: Biomaterials and Biomimetic Designs. Adv. Mater..

[93] Song K., Wang Z., Liu R., Chen G., Liu L. (2018). Microfabrication-Based Three-Dimensional (3-D) Extracellular Matrix Microenvironments for Cancer and Other Diseases. Int. J. Mol. Sci..

[94] Zhu Q., Hamilton M., Vasquez B., He M. (2019). 3D-printing enabled micro-assembly of a microfluidic electroporation system for 3D tissue engineering. Lab. Chip.

[95] Han X., Liu Z., Ma Y., Zhang K., Qin L. (2017). Cas9 Ribonucleoprotein Delivery via Microfluidic Cell-Deformation Chip for Human T-Cell Genome Editing and Immunotherapy. Adv. Biosyst..

[96] Hemmi H., Takeuchi O., Kawai T., Kaisho T., Sato S., Sanjo H., Matsumoto M., Hoshino K., Wagner H., Takeda K. (2000). A Toll-like receptor recognizes bacterial DNA. Nat. Cell Biol..

[97] Han X., Liu Z., Zhao L., Wang F., Yu Y., Yang J., Chen R., Qin L. (2016). Microfluidic Cell Deformability Assay for Rapid and Efficient Kinase Screening with the CRISPR-Cas9 System. Angew Chem..

[98] Byun S., Son S., Amodei D., Cermak N., Shaw J., Kang J.H., Hecht V.C., Winslow M.M., Jacks T., Mallicj P. (2013). Characterizing deformability and surface friction of cancer cells. Proc. Natl. Acad. Sci. USA.

[99] Zhang W., Kai K., Choi D.S., Iwamoto T., Nguyen Y.H., Wong H., Landis M.D., Ueno N.T., Chang J., Qin L. (2012). Microfluidics separation reveals the stem-cell-like deformability of tumor-initiating cells. Proc. Natl. Acad. Sci. USA.

[100] Hashimoto M., Takemoto T. (2015). Electroporation enables the efficient mRNA delivery into the mouse zygotes and facilitates CRISPR/Cas9-based genome editing. Sci. Rep..

[101] Geng T., Bao N., Sriranganathanw N., Li L., Lu C. (2012). Genomic DNA Extraction from Cells by Electroporation on an Integrated Microfluidic Platform. Anal. Chem..

[102] Bao N., Wang J., Lu C. (2008). Microfluidic electroporation for selective release of intracellular molecules at the single-cell level. Electrophoresis.

[103] Wang J., Bao N., Paris L.L., Wang H.-Y., Geahlen R.L., Lu C. (2008). Detection of Kinase Translocation Using Microfluidic Electroporative Flow Cytometry. Anal. Chem..

[104] Bian S., Zhou Y., Hu Y., Cheng J., Chen X., Xu Y., Liu P. (2017). High-throughput in situ cell electroporation microsystem for parallel delivery of single guide RNAs into mammalian cells. Sci. Rep..

[105] Zhang P., Zhang J., Bian S., Chen Z., Hu Y., Hu R., Li J., Cheng Y., Zhang X., Zhou Y. (2016). High-throughput superhydrophobic microwell arrays for investigating multifactorial stem cell niches. Lab. Chip.

[106] Szeto G.L., Van Egeren D., Worku H., Sharei A., Alejandro B., Park C., Frew K., Brefo M., Mao S., Heimann M. (2015). Microfluidic squeezing for intracellular antigen loading in polyclonal B-cells as cellular vaccines. Sci Rep..

[107] Li J., Wang B., Juba B.M., Vazquez M., Kortum S.W., Pierce B.S., Pacheco M., Roberts L., Strohbach J.W., Jones L.H. (2017). Microfluidic-Enabled Intracellular Delivery of Membrane Impermeable Inhibitors to Study Target Engagement in Human Primary Cells. ACS Chem. Biol..

[108] DiTommaso T., Cole J.M., Cassereau L., Buggé J.A., Hanson J.L.S., Bridgen D.T., Stokes B.D., Loughhead S.M., Beutel B.A., Kathrin Nussbaum G. (2018). Cell engineering with micro-fluidic squeezing preserves functionality of primary immune cells in vivo. Proc. Natl. Acad. Sci. USA.

[109] Kim E., Koo T., Park S.W., Kim D., Kim K., Cho H.-Y., Song D.W., Lee K.J., Jung M.H., Kim S. (2017). In vivo genome editing with a small Cas9 orthologue derived from Campylobacter jejuni. Nat. Commun..

[110] Müller M., Lee C., Gasiunas G., Davis T.H., Cradick T., Siksnys V., Bao G., Cathomen T., Mussolino C. (2016). Streptococcus thermophilus CRISPR-Cas9 Systems Enable Specific Editing of the Human Genome. Mol. Ther..

[111] Pawluk A., Amrani N., Zhang Y., Garcia B., Hidalgo-Reyes Y., Lee J., Edraki A., Shah M., Sontheimer E.J., Maxwell K.L. (2016). Naturally Occurring Off-Switches for CRISPR-Cas9. Cell.

[112] Harrington L.B., Paez-Espino D., Staahl B.T., Chen J.S., Ma E., Kyrpides N.C., Doudna J.A. (2017). A thermostable Cas9 with increased lifetime in human plasma. Nat. Commun..

[113] Hou Z., Zhang Y., Propson N.E., Howden S., Chu L.-F., Sontheimer E.J., Thomson J.A. (2013). Efficient genome engineering in human pluripotent stem cells using Cas9 from Neisseria meningitidis. Proc. Natl. Acad. Sci. USA.

[114] Joung J., Ladha A., Saito M., Segel M., Bruneau R., Huang M.W., Kim N.-G., Yu X., Li J., Walker B.D. (2020). Point-of-care testing for COVID-19 using SHERLOCK diagnostics. medRxiv.

[115] Qin P., Park M., Alfson K.J., Tamhankar M., Carrion R., Patterson J.L., Griffiths A., He Q., Yildiz A., Mathies R.A. (2019). Rapid and Fully Microfluidic Ebola Virus Detection with CRISPR-Cas13a. ACS Sens..

[116] Broughton J.P., Deng X., Yu G., Fasching C.L., Servellita V., Singh J., Miao X., Streithorst J.A., Granados A., Sotomayor-Gonzalez A. (2020). CRISPR–Cas12-based detection of SARS-CoV-2. Nat. Biotechnol..

[117] Rogacs A., Marshall L.A., Santiago J.G. (2014). Purification of nucleic acids using isotachophoresis. J. Chromatogr. A.

[118] Ramachandran A., Huyke D.A., Sharma E., Sahoo M.K., Banaei N., Pinsky B.A., Santiago J.G. (2020). Electric-field-driven micro-fluidics for rapid CRISPR-based diagnostics and its application to detection of SARS-CoV-2. Proc. Natl. Acad. Sci. USA.

[119] Schmidt G.W., Frey O., Rudolf F., Muzi-Falconi M., Brown G.W. (2017). The CellClamper: A Convenient Microfluidic Device for Time-Lapse Imaging of Yeast. Adv. Struct. Saf. Stud..

[120] Hass K., Bao M., He Q., Park M., Qin P., Du K. (2020). Integrated Micropillar Polydimethylsiloxane Accurate CRISPR Detection (IMPACT) System for Rapid Viral DNA Sensing. ACS Omega.

